# Exploring Public Interest in Multiple Sclerosis and Its Treatment Measures in the United States: A Google Trends Analysis

**DOI:** 10.7759/cureus.74649

**Published:** 2024-11-28

**Authors:** Zaed S Siddiqui, Vidith Phillips, Yusuf-Zain A Ansari, Jaskaran Singh, Sai K Angadala, Pruthvi Sai Chowdary Aluri, Chaitanya S Puvvada, Shreya Deoghare

**Affiliations:** 1 College of Science & Technology, Temple University, Philadelphia, USA; 2 Internal Medicine, Division of Biomedical Informatics and Data Science, Johns Hopkins University, School of Medicine, Baltimore, USA; 3 Neurology, Maharishi Markandeshwar University, Ambala, IND; 4 Neurology, Mamata Academy of Medical Sciences, Hyderabad, IND; 5 Neurology, Osmania General Hospital, Hyderabad, IND; 6 Internal Medicine, California Institute of Behavioral Neurosciences & Psychology, Fairfield, USA; 7 Department of Dermatology, Dr. D. Y. Patil Medical College, Hospital & Research Centre, Pimpri-Chinchwad, IND

**Keywords:** beta interferon, disease modifying therapy, google trends, multiple sclerosis, plasmapheresis

## Abstract

Introduction

Multiple sclerosis (MS) afflicts over 2.8 million individuals worldwide and is a leading cause of neurological impairment in young adults. This study investigates the public interest in MS and its treatment options in the United States over the past decade, utilizing Google Trends data. The aim is to analyze how search trends reflect public engagement with MS, particularly about managing relapses, slowing disease progression, alleviating symptoms, and enhancing quality of life.

Methods

A cross-sectional study was conducted utilizing Google Trends to analyze search interest related to MS and its treatments from January 2014 to December 2023. The data was divided into two five-year periods: 2014-2018 and 2019-2023. Specific search terms for MS and various treatment modalities were analyzed, including traditional therapies (e.g., interferon-beta, glatiramer acetate) and newer treatments (e.g., ocrelizumab, siponimod). Statistical analysis was performed using the Mann-Whitney U test to compare relative search volumes (RSV) between the two periods.

Results

Significant fluctuations in RSV were observed over the study period. A notable increase in RSV was found for treatments such as rituximab, ocrelizumab, ublituximab, siponimod, and ponesimod in the 2019-2023 period compared to 2014-2018 (p < 0.05). Conversely, a significant decrease in RSV was observed for glatiramer acetate, alemtuzumab, natalizumab, fingolimod, and plasmapheresis during the same periods (p < 0.05). Treatments like beta interferon, ofatumumab, and teriflunomide showed no significant change in RSV between the two periods (p > 0.05).

Conclusion

Dividing the search period into two five-year intervals revealed shifting public interest toward newer MS therapies over the past decade. The increased interest in recent treatments aligns with advancements in MS management and may influence patient inquiries and treatment decisions. These findings highlight the utility of Google Trends as a tool for monitoring public awareness and underscore the importance of providing accessible, accurate information to guide healthcare strategies and policymaking.

## Introduction

Multiple sclerosis (MS) is a chronic, often debilitating neurological disorder that affects more than 2.8 million individuals worldwide, making it a leading cause of neurological impairment in young adults [1,2]. The unpredictable nature of MS, characterized by periods of relapse and remission, imposes significant physical, cognitive, and psychological burdens on patients, thereby profoundly impacting their quality of life [2]. Understanding how public interest in MS and its treatments evolves is essential for aligning patient education efforts and healthcare strategies with emerging trends [3].

Over the years, the treatment of MS has evolved significantly. Traditional management includes disease-modifying therapies (DMTs) such as interferon-beta and glatiramer acetate, which reduce relapse frequency and slow disability progression [4]. However, in the past decade, newer therapies-including monoclonal antibodies like ocrelizumab and oral agents such as fingolimod and dimethyl fumarate have demonstrated improved efficacy [5]. Furthermore, recent research into stem cell therapy and neuroprotective agents offers promising avenues for future treatment options [6].

As these advancements emerge, public awareness and interest in MS treatments play a crucial role in shaping patient engagement and adherence to therapies. Assessing these trends is particularly valuable for identifying gaps in understanding and optimizing communication strategies [3]. In the digital age, Google Trends has become a valuable tool in healthcare info epidemiology, enabling insights into public interest in various health-related topics [7]. Analyzing relative search volume (RSV) rather than absolute search counts, Google Trends allows researchers to track and compare changes in interests in specific search terms over time, offering a unique perspective on the public's awareness and concerns regarding diseases like MS and their treatments [7].

The purpose of this study is to elucidate how public interest in MS and its treatment modalities has evolved in the United States over the past decade, using Google Trends data. While treatment decisions are ultimately made by healthcare providers, understanding public interest can help healthcare professionals and policymakers tailor educational initiatives and communication strategies to address patient concerns and improve adherence to recommended therapies [3].

## Materials and methods

A cross-sectional study was conducted over one week from July 16 to July 23, 2024. As no human participants were involved and only publicly available data sources were used, ethics committee approval was not required by standard research guidelines.

Google Trends, a public web facility of Google Inc., was utilized to analyze the popularity of top search queries in Google Search across various regions and languages. This tool provides graphical representations to compare the search volume of different queries over time, allowing exploration of topics, search terms, and trending data. It offers insights into how search interest evolves globally and locally [6]. The key metric used was RSV, which quantifies the frequency of a specific search term relative to the total search volume within a defined period and region. RSV is expressed on a scale from 0 to 100, indicating the term's popularity compared to its peak search volume during the timeframe. This normalized measure facilitates comparison across different terms and periods. While RSV was the primary metric used in this study, other parameters such as absolute search volume and geographical distribution data could also be analyzed, though these were not included in this study due to data access limitations.

For this study, data was collected from the United States, focusing on search trends from January 2014 to December 2023. The keyword “multiple sclerosis” was used to assess overall interest in the disease. To explore public interest in treatment options, specific search terms corresponding to various treatment modalities were individually analyzed and categorized as follows: beta interferon, glatiramer acetate, ofatumumab, alemtuzumab, natalizumab, rituximab, ocrelizumab, ublituximab, fingolimod, siponimod, ponesimod, teriflunomide, Methylprednisolone, plasmapheresis, and intravenous immunoglobulin.

Data obtained from Google Trends was exported to Microsoft Excel for organization and preliminary analysis. Statistical analyses were performed using R version 4.3.2 (R Core Team, 2023), a comprehensive statistical software environment. Trends in the RSV for MS over the study period were visualized using line graphs to illustrate fluctuations over time. For the analysis of RSV related to treatment modalities, the data was divided into two five-year periods: January 2014 to December 2018 and January 2019 to December 2023. The Mann-Whitney U test, a non-parametric test appropriate for comparing two independent groups without assuming normal distribution, was employed to assess the statistical significance of changes in RSV between these periods. A p-value of less than 0.05 was considered statistically significant.

## Results

**Figure 1:**
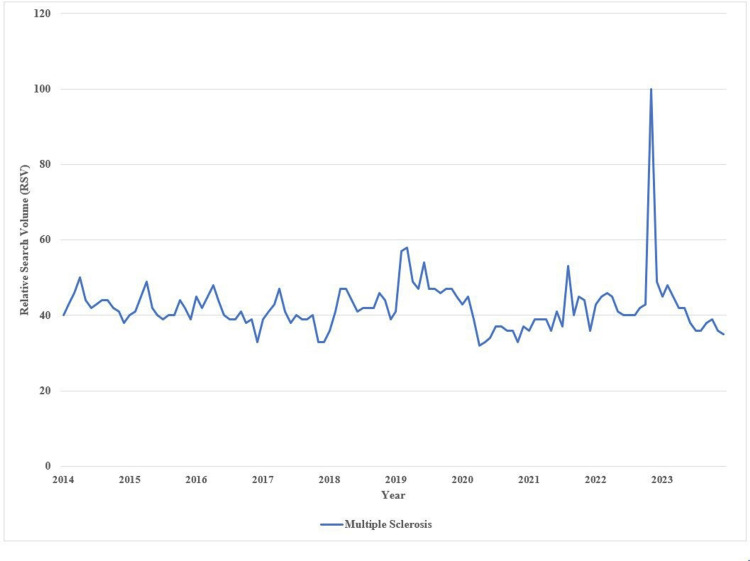
Changes in the RSV of Multiple Sclerosis from 2014 to 2023 in the United States RSV: relative search volume

The analysis of Google Trends data revealed notable fluctuations in the RSV for “Multiple Sclerosis” in the United States from January 2014 to December 2023. During the initial period (2014-2018), the RSV generally remained in the 40s, with minor variations. The lowest RSV was recorded in December 2016 (RSV of 34), while the highest peaks occurred in April 2014 and April 2015 (RSV of 53). From January 2019 onward, more pronounced fluctuations were observed, with significant spikes in February 2019 (RSV of 61) and November 2022 (RSV of 100). Toward the end of 2023, the RSV declined, reaching a low of 37 in December. Figure 1 illustrates the changes in the RSV of MS over the years in the United States, providing a visual representation of the trends observed.

To assess changes in public interest for specific MS treatment modalities, we compared the RSV between two five-year periods: January 2014 to December 2018 and January 2019 to December 2023. The normality of the variables was assessed using the Shapiro-Wilk test, which indicated that the data did not follow a normal distribution. Consequently, the Mann-Whitney U test, a non-parametric statistical method, was chosen because it does not assume normality and is appropriate for comparing two independent groups. This test evaluates whether the distributions of RSV values between the two periods differ significantly. A p-value of less than 0.05 was considered statistically significant.

Table 1 summarizes the distribution of search frequencies for specific treatment terms across the two periods. The results indicate that RSV for most terms significantly differed between the two time frames.

**Table 1:** Distribution of Search Frequency for Specific Treatment Terms Across Two Five-Year Periods *P-values <0.05 have been considered statistically significant.

Terms	From January 2014 to December 2018 (Median (IQR))	From January 2019 to December 2023 (Median (IQR))	P-values	Z-values
Disease-Modifying Therapy				
Beta Interferon	0.00 (0.00 – 0.00)	0.00 (0.00 – 0.00)	0.317	-0.091
Glatiramer Acetate	70.00 (63.00 – 84.75)	47.50 (37.50 – 54.25)	<0.000*	1.374
Ofatumumab	42.00 (32.25 – 53.00)	37.50 (31.25 – 43.75)	0.108	3.778
Alemtuzumab	52.00 (49.00 – 58.50)	27.00 (22.25 – 32.00)	<0.000*	1.170
Natalizumab	78.50 (73.00 – 84.75)	60.00 (54.25 – 64.00)	<0.000*	1.697
Rituximab	69.50 (63.25 – 80.75)	81.50 (75.00 – 85.00)	<0.000*	-1.780
Ocrelizumab	12.00 (2.00 – 51.75)	64.00 (60.00 – 68.00)	<0.000*	-1.560
Ublituximab	0.00 (0.00 – 0.00)	25.50 (0.00 – 32.75)	<0.000*	-0.724
Fingolimod	81.00 (73.50 – 87.00)	49.00 (42.00 – 66.25)	<0.000*	0.723
Siponimod	0.00 (0.00 – 8.00)	34.50 (25.25 – 50.25)	<0.000*	-0.873
Ponesimod	0.00 (0.00 – 0.00)	12.50 (0.00 – 43.75)	<0.000*	-0.529
Teriflunomide	75.00 (65.25 – 83.75)	70.00 (62.50 – 84.00)	0.350	-0.401
Relapse Management				
Methylprednisolone	57.00 (54.00 – 66.00)	80.50 (72.00 – 89.00)	<0.000*	-1.207
Plasmapheresis	79.00 (74.00 – 84.75)	74.00 (69.00 – 79.00)	0.003*	0.504

A significant increase in RSV was observed for treatments such as rituximab, ocrelizumab, ublituximab, siponimod, ponesimod, and methylprednisolone in the last five years compared to the previous five years (p < 0.05). This upward trend suggests growing public interest in these therapies, possibly due to their recent approval, increased clinical adoption, or emerging research demonstrating their efficacy.

Conversely, a significant decrease in RSV was noted for glatiramer acetate, alemtuzumab, natalizumab, fingolimod, and plasmapheresis during the same period (p < 0.05). The decline in interest in these treatments may reflect a shift toward newer therapeutic options or a perceived reduction in their relevance or effectiveness over time.

For treatments like beta interferon, ofatumumab, and teriflunomide, the RSV remained relatively stable, with no significant differences observed between the two periods (p > 0.05). This stability suggests consistent public interest and awareness of these therapies without major shifts over the past decade.

## Discussion

The results of this study offer valuable insights into the changing public interest in MS and its treatment modalities over the past decade, as reflected by Google Trends data. The fluctuations in RSV observed for specific MS treatments indicate evolving public and clinical interest in these therapies.

The findings of this study align with previous studies that utilized Google Trends to assess public interest in various health-related topics, highlighting the utility of Google Trends as a proxy for gauging public awareness and behavior in the context of chronic diseases [8,9]. For instance, the significant increase in RSV for drugs such as ocrelizumab, rituximab, and methylprednisolone during the period of 2019-2023 is consistent with their increased clinical adoption and growing evidence supporting their efficacy in managing MS [10,11]. Ocrelizumab has been noted for its effectiveness in both relapsing-remitting and primary progressive forms of MS, which may explain the heightened public interest observed in this study [5].

The decline in RSV for therapies like glatiramer acetate, alemtuzumab, and fingolimod could reflect a shift in therapeutic preferences toward newer, more efficacious treatments [12,13]. This trend aligns with a broader paradigm shift in MS management, where higher-efficacy DMTs are increasingly utilized earlier in the disease course to improve long-term outcomes, as highlighted by recent studies [13,14]. The stability in RSV for beta interferon, ofatumumab, and teriflunomide suggests that while these treatments remain relevant, they have not experienced the same surge in interest as other therapies. This could be due to their established role in the treatment landscape, with less novel information being generated or fewer ground-breaking studies being published in recent years [15].

Moreover, the increased interest in treatments such as siponimod and ponesimod, which are relatively newer entrants to the MS treatment arsenal, aligns with recent studies highlighting their potential benefits and the need for continuous monitoring of their long-term efficacy and safety [16]. The rise in RSV for these medications suggests that the public is increasingly seeking information about emerging therapies, possibly reflecting a broader awareness of the advancements in MS treatment options.

While this study provides valuable insights into public interest trends in MS treatments, several limitations should be considered. First, Google Trends data represents search behavior, which may not directly correlate with treatment utilization or clinical outcomes. The RSV is influenced by multiple factors, including media coverage, health campaigns, and even specific events that may temporarily skew search volumes. Therefore, caution should be exercised in interpreting these trends as direct indicators of treatment popularity or clinical efficacy. Second, the study is limited to search data from the United States, which may not be generalizable to other regions or countries with different healthcare systems, treatment availability, or cultural perceptions of MS. The selection of search terms and their categorization into specific treatment modalities may also influence the results, as different search queries might capture varying levels of public interest. Third, the use of Google Trends as a data source is inherently limited by the platform's algorithms and data processing methods, which are not fully transparent. The study's reliance on RSV rather than absolute search counts further complicates the interpretation, as it reflects proportional rather than absolute changes in public interest. Fourth, Google Trends does not provide detailed demographic information about the individuals conducting the searches. As such, it is not possible to determine whether the search behavior represents patients, caregivers, or healthcare providers, nor is it possible to analyze how age, gender, socioeconomic status, or other demographic factors might influence search trends. Addressing these gaps in future research could provide a more nuanced understanding of public interest in MS treatments.

## Conclusions

This study reveals significant fluctuations in public interest regarding MS and its treatment modalities over the past decade, as evidenced by Google Trends data. The increasing RSV for newer therapies such as ocrelizumab, rituximab, and methylprednisolone indicates a growing public interest aligned with recent advancements and clinical adoption of these treatments, while the declining interest in older therapies like glatiramer acetate and fingolimod suggests a shift toward more effective or recently approved options. These findings demonstrate the utility of Google Trends as a valuable tool for monitoring public awareness and can help healthcare providers, researchers, and policymakers understand which treatments are gaining attention and potentially influencing patient inquiries and treatment decisions. This kind of data can be leveraged to design targeted public health campaigns that address knowledge gaps and misconceptions about MS treatments. Additionally, the efficacy of such campaigns can be evaluated using Google Trends. Integrating these insights into patient-centered communication strategies could ultimately improve adherence to treatment plans and enhance patient outcomes.
